# Metabolomics data of *Mitragyna speciosa* leaf using LC-ESI-TOF-MS

**DOI:** 10.1016/j.dib.2018.04.001

**Published:** 2018-04-04

**Authors:** Rubashiny Veeramohan, Kamalrul Azlan Azizan, Wan Mohd Aizat, Hoe-Han Goh, Sharif Mahsufi Mansor, Nur Sabrina Mohd Yusof, Syarul Nataqain Baharum, Chyan Leong Ng

**Affiliations:** a Institute of Systems Biology (INBIOSIS), Universiti Kebangsaan Malaysia (UKM), 43600 Bangi, Selangor, Malaysia; b Centre for Drug Research, Universiti Sains Malaysia, 11800 Minden, Penang, Malaysia

**Keywords:** Alkaloids, LC–MS, Metabolomics, Methanolic extraction, *Mitragyna speciosa*

## Abstract

*Mitragyna speciosa* is a psychoactive plant known as “ketum” in Malaysia and “kratom” in Thailand. This plant is distinctly known to produce two important alkaloids, namely mitragynine (MG) and 7-hydroxymitragynine (7-OH-MG) that can bind to opioid receptors [1]. MG was reported to exhibit antidepressant properties in animal studies [2]. These compounds were also proposed to have the potential to replace opioid analgesics with much lower risks of side effects [3]. To date, there are only over 40 metabolites identified in *M. speciosa* [4,5]. To obtain a more complete profile of secondary metabolites in ketum, we performed metabolomics study using mature leaves of the green *M. speciosa* variety. The leaf samples were extracted using methanol prior to liquid chromatography-electrospray ionization-time of flight-mass spectrometry (LC-ESI-TOF-MS) analysis. This data can be useful to for the identification of unknown metabolites that are associated with alkaloid biosynthesis pathway in *M. speciosa*.

**Specifications**

**Table:** 

Subject area	Biology
More specific subject area	Metabolomics
Type of data	Analyzed LC-ESI-TOF-MS Data
How data was acquired	Raw data attained from MicroTOF-Q III (Bruker Daltonic) using an ESI positive ionization coupled with Ultimate 3000 UHPLC system (Dionex)
Data format	Analyzed data in the form of.xlsx file
Experimental factors	Methanolic extracts from mature leaves of *M. speciosa* of the green variety
Experimental features	Data was processed using ProfileAnalysis 2.1
Data source location	Bangi, Malaysia
Data accessibility	Supplementary Table 1

**Value of the data**

• LC-MS data allow metabolite profiling and the identification of target metabolite compounds present in *M. speciosa* leaf.
• The data will also be useful in the reconstruction of secondary metabolite biosynthesis pathway in *M. speciosa*.
• Metabolomics analysis will provide insights on the metabolite expression in mature leaf of *M. speciosa*.

## 1 Data

*Mitragyna speciosa* (*M. speciosa*) is a tropical plant known to produce the alkaloids mitragynine (MG) and 7-hydroxymitragynine (7-OH-MG) that bind to the G-protein-coupled mu-opioid receptor [1]. MG exhibits antidepressant properties in mice [2]. The compounds were also proposed to potentially replace opioid analgesics with notably lesser risks of side effects [3]. There are only over 40 metabolites identified in *M. speciosa* thus far [4], [5]. To attain a more complete profile of secondary metabolites, here we report the metabolomics dataset from the mature leaves of green *M. speciosa* variety. This dataset shows the retention time (RT), mass-to-charge (*m/z*) values of compounds detected in mature leaves of the green variety of *M. speciosa*, and normalized peak intensity values of five biological replicates, each with five technical replicates. The raw data was obtained from mass spectrometry (MS) analysis and was processed using ProfileAnalysis 2.1 (Bruker). The processed data is provided in Microsoft Excel (.xlsx) file (Supplementary Table 1).

## 2 Experimental design, materials and methods

### 2.1 Chemicals and reagents

Analytical-grade methanol (CH_3_OH) was purchased from Merck, Germany. Umbelliferone (C_9_H_6_O_3_, purity 99%) was purchased from Sigma-Aldrich, USA.

### 2.2 Sample preparation

Mature leaf samples of *M. speciosa* were flash frozen using liquid nitrogen, supplied by Universiti Sains Malaysia. The frozen leaves were stored in − 80 °C freezer prior to metabolite extraction.

### 2.3 Metabolite extraction

Sample extraction was done based on [6] with slight modifications. Mature leaves were individually ground with mortar and pestle in liquid nitrogen, weighed and transferred into respective Falcon tubes. Freshly prepared ice cold methanol (5 mL) was added to 100 mg of powdered samples, immediately vortexed and incubated on dry ice for 8 h prior to 20 °C overnight incubation in a high capacity incubator shaker. The samples were then centrifuged at 6000 rpm for 10 min at 4 °C. The supernatant was collected and filtered with 0.2 µm polytetrafluoroethylene (PTFE) syringe filter. Next, 1 mL of samples were transferred into sample vials and stored in − 80 °C freezer to avoid degradation. Prior to LC-TOF-MS analysis, the samples were spiked with 100 ppm of umbelliferone as an internal standard. A total of five biological replicates were prepared from individual leaf samples.

### 2.4 Liquid chromatography–mass spectrometry (LC–MS)

Chromatographic separation of samples was performed using Thermo Scientific C18 column (Acclaim^™^ Polar Advantage II, 3 × 150 mm, 3 µm particle size) with an Ultimate UHPLC system (Dionex). Gradient elution was performed at 0.4 mL/min and 40 °C using 0.1% formic acid in water (A) and 100% acetonitrile (ACN) (B) as mobile phases with a total run time of 15 min. The injection volume of sample was 1 µL and the gradient started at 5% B (0–0.5 min); followed by 90% B (0.5–6 min); 90% B (6–10 min); 5% B (10–12 min); and 5% B (12–15 min). High resolution mass spectrometry was carried out using a MicroTOF-Q III Bruker Daltonic using an ESI positive ionization with the settings of 4500 V capillary voltage; 1.2 bar nebulizer pressure; and 8 L/min at 200 °C drying gas. The mass range was 50 to 1000 *m/z*. Five technical runs were performed for each of the five biological replicates.

### 2.5 Data processing

The accurate mass data of molecular ions, provided by the TOF analyzer, were processed by Compass Data Analysis software (Bruker Daltonik GmbH). The MS raw dataset obtained was prepared using ProfileAnalysis 2.0 (Bruker Daltonic, Germany) for data bucketing and then converted to.xlsx format for further statistical analysis. Find molecular features (FMF) and retention time alignment were used to mine the LC-ESI-TOF-MS data and to attain relevant rt-*m/z* pairs [7]. The FMF algorithm defines compounds in a sample by linking retention time, mass, and intensity. The consequential compound data was then generated as a bucket table. Each bucket stands for one compound (peak). Hence, each bucket represents one feature (RT:*m/z*) in the bucket table (Supplementary Table 1).

### 2.6 Metabolite identification

The identification of several metabolites in this LC–MS analysis (Table 1) was achieved through mass-based search followed by manual verification [8]. The *m/z* value of a molecular ion of interest was searched against literature and online databases, namely MetFrag [9] and METLIN [10]. Metabolites with molecular weights within a specified tolerance range to the query *m/z* value were retrieved from the databases as putative identification. The *m/z* values that correspond to several previously reported alkaloids, including mitragynine, 7-hydroxymitragynine, mitragynaline, and paynantheine were successfully identified. However, one mass may link to different elemental composition, or even similar composition but different structures. Hence, some *m/z* values will have more than one putative compounds (Table 1). Since LC–MS alone is incapable of distinguishing isomers, further identification using tandem mass spectrometry (MS/MS) will need to be carried out for confirmation of these compounds.

**Table 1:** Putative identification of metabolites in mature *Mitragyna speciosa* leaf.

**Retention time (min)**	***m/z* [M+H]**^**+**^	**Putative compound(s)**	**Elemental composition**	**Source**
4.67	399.126	Caulerpin	C24H18N2O4	MetFrag
4.77	355.199	Yohimbine	C21H26N2O3	MetFrag
				
5.07	401.205	Isospeciofoline	C22H28N2O5	Avula et al. [11]
		Isorotundifoline	C22H28N2O5	
				
5.14	385.210	Corynoxine	C22H28N2O4	Avula et al. [11]
		Corynoxine B		
				
5.18	415.221	7-hydroxymitragynine	C23H30N2O5	Avula et al. [11]
		7β-hydroxy-7H-mitraciliatine	C23H30N2O5	
				
5.43	397.211	Paynantheine	C23H28N2O4	Avula et al. [11]
		3-Isopaynantheine		
				
9.09	399.268	Mitragynine	C23H30N2O4	Avula et al. [11]
		Speciogynine		
		Speciociliatine		
				
9.77	380.333	Mitragynaline	C21H20N2O5	Houghton et al. [12]
5.58	350.060	Corynantheidaline	C20H18N2O4	Houghton et al. [12]
